# STAT3 signal transduction through interleukin-22 in oral squamous cell carcinoma

**DOI:** 10.3892/ijo.2012.1594

**Published:** 2012-08-21

**Authors:** LUTFUN NAHER, TAMOTSU KIYOSHIMA, IEYOSHI KOBAYASHI, HIROKO WADA, KENGO NAGATA, HIROAKI FUJIWARA, YUKIKO F. OOKUMA, SATORU OZEKI, SEIJI NAKAMURA, HIDETAKA SAKAI

**Affiliations:** 1 Laboratory of Oral Pathology and Medicine, Division of Maxillofacial Diagnostic and Surgical Sciences, Faculty of Dental Science, Kyushu University;; 2 Department of Oral and Maxillofacial Oncology, Division of Maxillofacial Diagnostic and Surgical Sciences, Faculty of Dental Science, Kyushu University;; 3 Department of Pediatric Dentistry, Division of Oral Health, Growth and Development, Faculty of Dental Science, Kyushu University, Fukuoka 812-8582;; 4 Section of Oral Surgery, Department of Oral and Maxillofacial Surgery, Fukuoka Dental College, Fukuoka 814-0193, Japan

**Keywords:** IL-22, squamous cell carcinoma, STAT3, cell differentiation

## Abstract

Interleukin (IL)-22 is a member of the IL-10 family. Its main targets are epithelial cells, not immune cells. We examined IL-22 signal transduction in oral squamous cell carcinoma (OSCC) cells. Immunohistochemical staining revealed that IL-22R was expressed more highly in OSCC compared to normal regions. An IL-22R signal was also observed in metastatic OSCC cells in the lymph node. RT-PCR showed that the human OSCC cell lines MISK81-5, HSC-3, HSC-4, SAS and SQUU-B expressed IL-22 receptor chains. Immunoblotting showed that IL-22 induced a transient tyrosine phosphorylation of STAT3 (pY705-STAT3) in MISK81-5 cells. The change in the serine phosphorylation of STAT3 was subtle during the examination periods. Simultaneously, pY705-STAT3 activation in HSC-3 cells was undetectable after IL-22 stimulation. Immunocytochemistry demonstrated that IL-22 induced the translocation of phosphorylated STAT3 into the nucleus of MISK81-5 cells. IL-22 temporarily upregulated the expression of anti-apoptotic and mitogenic genes such as Bcl-x, survivin and c-Myc, as well as SOCS3. IL-22 transiently activated ERK1/2 and induced a delayed phosphorylation of p38 MAP kinase, but negligibly involved the activation of NF-κB in MISK81-5 cells. MISK81-5 and SQUU-B cells treated with IL-22 showed mild cellular proliferation. MISK81-5, HSC-4 and SAS cells treated with IL-22 downregulated the keratinocyte differentiation-related genes compared with unstimulated cells. Conversely, STAT3 suppression by STAT3 siRNA strongly disrupted the down-regulation of these genes by IL-22, but it did not significantly affect the activation of ERK1/2 by IL-22. The OSCC cells used in this study upregulated the expression of SERPINB3/4 (SCCA1/2), well-known SCC markers, following treatment with IL-22. These results indicate that IL-22 differentially activates the STAT3 signaling system depending on the type of OSCC. IL-22 may therefore play a role in tumor growth, cell differentiation and progression through STAT3-dependent and -independent pathways.

## Introduction

More than 90% of all malignant epithelial tumors arising in the oral cavity are squamous cell carcinomas (SCC)(1,2). Oral squamous cell carcinoma (OSCC) is one of the most common malignancies in humans. However, the overall survival rates have not substantially improved for decades (3) and a significantly increased incidence of OSCC in young subjects has been reported in recent decades (4,5).

In general, varying degrees of inflammatory cell infiltration are observed around malignant tumors containing SCC. Cytokines or cytokine-related mediators have direct proliferative and anti-proliferative effects on tumor cells, and influence the cellular behavior of malignant cells (6–8). Interleukin (IL)-22 is a newly discovered member of the IL-10 family, and is expressed mainly in activated T, mast and NK cells (9,10). Additionally, a subset of helper T cells abundantly produces IL-22, suggesting it plays a significant role in skin homeostasis and pathology (11). IL-22 receptor is a heterodimeric receptor of class II cytokine consisting of two chains, IL-22R1 and IL-10R2. IL-22R1 is expressed in non-immune tissues, including the skin, lungs, small intestine, liver, colon, kidneys, and pancreas (12), unlike IL-10R2, which is ubiquitously expressed in various organs and cells. Since IL-22 does not act between immune cells, but rather from immune cells to the non-immune cell compartment, IL-22 appears to be unique among cytokines.

Although a few studies have so far addressed the roles of IL-22 in malignant cell proliferation and apoptosis, there are inconsistencies in the findings. IL-22 induces the activation of the major MAPK pathways in hepatoma cells (13), and increases the expression of many anti-apoptotic and mitogenic proteins following the activation of STAT3 (14). IL-22 can accelerate inducible nitric-oxide synthase expression in human colon adenocarcinoma cells (15). IL-22 protects human lung non-small cell carcinoma cells against chemotherapy via the activation of anti-apoptotic proteins (16). Conversely, IL-22 treatment induces the cell cycle arrest of murine breast adenocarcinoma EMT6 cells through the inhibition of ERK1/2 and AKT phosphorylation (17). The survival of mice with IL-22-expressing Colon 26 cells significantly increased in comparison to the control mice, suggesting that IL-22 might play a protective role in hosts with tumors (18). Although IL-22 appears to act variously in different carcinoma cells, there is little knowledge on the potential roles of IL-22 in OSCCs.

This study analyzed the signal transduction and genes induced in OSCC cells, to comprehensively evaluate the potential biological activity of IL-22 in OSCC. Additionally, the cell differentiation of OSCC cells by IL-22 was examined.

## Materials and methods

### Reagents

Recombinant human IL-6, IL-22 and TNF-α (Wako, Osaka, Japan), and mouse EGF (Sigma-Aldrich, St. Louis, MO, USA) were used for the study. Antibodies reactive to total STAT3, phospho-STAT3 (pY705, pS727), ERK1, phospho-ERK1/2 (pT202/pY204), p38α and phospho-p38 (pT180/pY182) were purchased from BD Biosciences (Franklin Lakes, NJ, USA). The antibodies for total STAT3 (Santa Cruz Biotechnology, Inc., Santa Cruz, CA, USA) and IL-22R (Novus Biologicals, Littleton, CO, USA) were used for the immunocytochemical and immunohistochemical studies, respectively.

### Samples and immunohistochemistry

Samples of primary OSCC and metastatic OSCC in the cervical lymph node diagnosed in the Department of Oral and Maxillofacial Surgery, Kyushu University Hospital in 2011 were immunostained in this study. This study was approved by the local research ethics committee.

Immunohistochemical staining was performed on 5 *μ*m paraffin sections. The endogenous peroxide activity was eliminated by treatment with 3% hydrogen peroxide in methanol for 20 min. Non-specific protein binding was blocked with 10% goat serum for 20 min, and then the sections were reacted with the primary antibody at 4°C overnight. The sections were incubated with the Fab’ fragment of the secondary antibody conjugated with a peroxidase-labeled amino acid polymer (Histofine Simple Stain MAX PO, Nichirei, Japan) for 30 min at room temperature. After washing with PBS, the immunoreactivity was visualized with a solution of 3, 3′-diaminobenzidine and <0.1% hydrogen peroxide (Nichirei). Subsequently, the sections were counterstained with hematoxylin. For the negative control, PBS was substituted for the primary antibody.

### Cell lines and culture conditions

Human OSCC cell lines, MISK81-5 (19), HSC-3, HSC-4 (Japanese Cancer Research Resources Bank), SAS (20), and SQUU-B (21), a human keratinocyte cell line, HaCaT, and a human erythroleukemia cell line, K562, were used. MISK81-5, HSC-3, HSC-4 cells and K562 cells were grown in α-MEM (Invitrogen, Carlsbad, CA, USA) with 10% fetal bovine serum (Filtron, Brooklyn, Australia). SAS and SQUU-B cells were incubated in DMEM/F-12 (Invitrogen) with 10% serum. HaCaT cells were maintained in DMEM (Invitrogen) with 10% serum.

### Semiquantitative RT-PCR and real-time quantitative PCR analyses

The total RNAs were isolated using the SV Total RNA Isolation System (Promega, Madison, WI, USA), and cDNAs were generated from isolated total RNA with the SuperScript VILO cDNA Synthesis Kit (Invitrogen). Semiquantitative PCR was amplified with Advantage 2 (Clontech, Mountain View, CA, USA). Real-time quantitative PCR was performed using a Thermal Cycler Dice^®^ Real Time System with SYBR^®^ Premix Ex Taq™ II (Takara, Shiga, Japan).

A reference gene was determined among the various housekeeping genes (Table I). The relative expression level of each targeted gene was normalized using the ΔΔC_T_ comparative method, based on the reference gene threshold cycle (CT) values (22).

The mRNA expression of the STAT3 downstream genes, keratinocyte differentiation-related genes and SERPINB3/4 (Squamous Cell Carcinoma Antigen, SCCA1/2) genes, well-known SCC markers, were examined in OSCC cells after IL-22 stimulation (Table I). The specificity of the PCR products was determined using a melting curve and/or gel electrophoresis.

### Immunoblotting

Proteins were separated by 12% SDS-polyacrylamide gel electrophoresis, and transferred to an Immun-Blot^®^ PVDF Membrane (Bio-Rad, Hercules, CA, USA). Antibodies bound to proteins were visualized by the ECL plus detection system (Amersham, Piscataway, NJ, USA). The protein concentration was estimated using a Micro BCA Protein Assay Kit (Pierce Biotechnology, Inc., IL, USA).

### Immunocytochemistry for STAT3

Following incubation with the primary antibody, the cells were incubated in Alexa Fluor^®^ 568 goat anti-rabbit IgG or 594 rabbit anti-mouse IgG (Invitrogen). The nuclei were counterstained with DAPI (Dojindo, Kumamoto, Japan).

### Cell proliferation assay

The proliferation of IL-22-treated cells was quantified using the CellTiter-Glo^®^ Luminescent Cell Viability Assay (Promega) and a Microplate Luminometer (Turner Biosystems, Sunnyvale, CA, USA). The cells were stimulated with 20 ng/ml of IL-22 every 24 h during the 48 h culture period.

### Construction of an NF-κB-responsive Luciferase Reporter Vector and the luciferase assay

Four tandem copies of the NF-κB consensus sequence were inserted upstream of the minimal promoter (minP) in pGL4.26 [*luc2*/minP/Hygro] (Promega). After clonal selection of stably transfected MISK81-5 cells with hygromycin, MISK-pGL4-NF-κB cells were generated. Luminescence was measured using the One-Glo luciferase system (Promega) and the Microplate Luminometer.

### Transient transfection of siRNA for STAT3

siRNAs for human STAT3 (GenBank Accession Number: NM_003150) and GAPDH, and a siRNA universal negative control (Sigma-Aldrich) were used as a target and positive and negative controls, respectively. The cells were transfected with siRNA (10 nM) using the Lipofectamine RNAiMAX (Invitrogen).

### Statistical analysis

All experiments were independently repeated at least three times. Statistical analysis was performed using the one-way ANOVA with the Tukey-Kramer comparison test, Dunnett’s test or Student’s t-test. A p-value <0.05 or <0.01 was considered to indicate statistically significant differences.

## Results

### Human oral squamous cell carcinoma cell lines express IL-22 receptor chains

**Figure 1.**
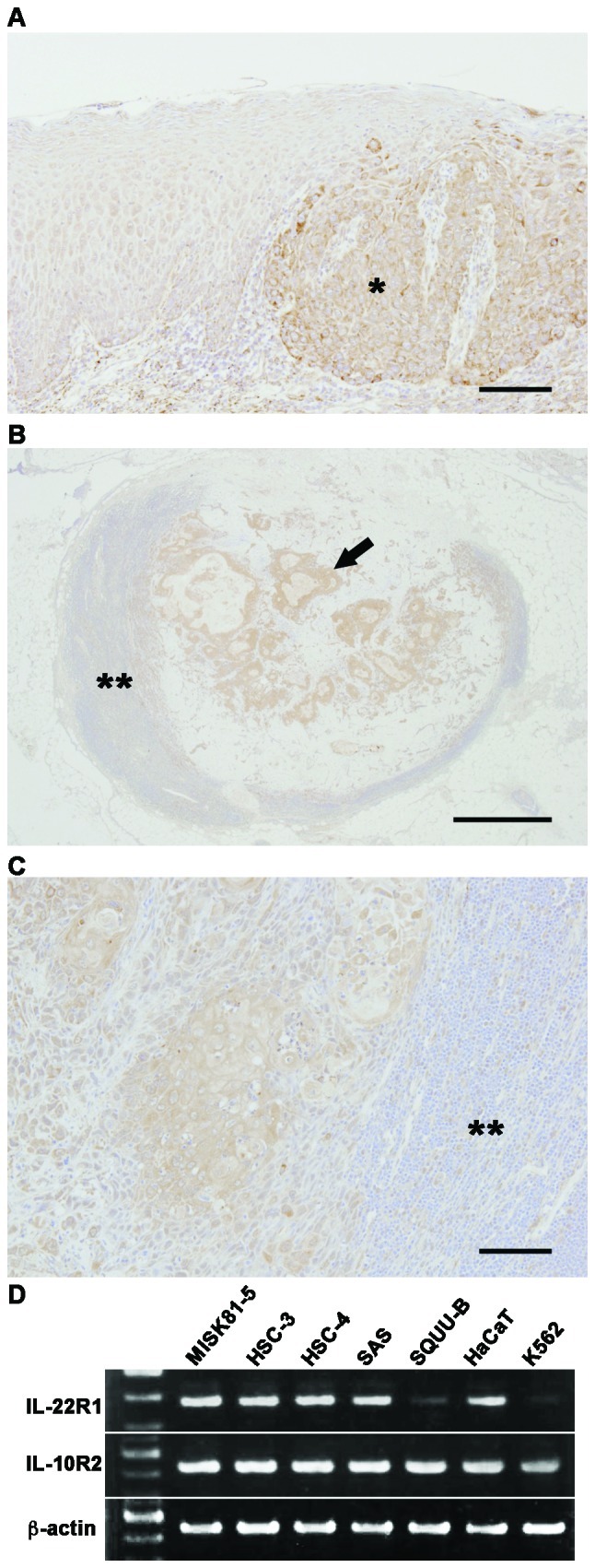
IL-22R is expressed in the OSCC of the extirpated samples and OSCC cell lines. (A) IL-22R immunostaining was observed in four of the seven extirpated samples, including some normal regions. The immunoreactive intensity for IL-22 was increased in the OSCC region (right side; *) compared with that in the normal oral epithelium (left side). Scale bar, 100 *μ*m. (B) Metastatic carcinoma cells in the cervical lymph node (**) showed strongly positive signals for IL-22R (arrow). Scale bar, 1 mm. (C) IL-22R positive signals in metastatic carcinoma cells in the cervical lymph node (**) in another case. Scale bar, 100 *μ*m. (D) IL-10R2 and IL-22R1 mRNA expressions were examined by semiquantitative RT-PCR. β-actin was used as an internal control.

First, we immunohistochemically examined the IL-22R expression in OSCC. The immunostaining revealed that the intensity increased in the OSCC cells, although weak IL-22R signals were also observed throughout the normal oral mucosa (Fig. 1A). Significant staining was also observed in the metastatic carcinoma cells present in the cervical lymph node (Fig. 1B and C).

IL-22R1 and IL-10R2 were both detectable in all tested OSCC cells (Fig. 1D), although their expression intensity varied. HaCaT cells served as a positive control (23). K562 cells were analyzed as a negative control for IL-22R1. The mRNA expression of IL-22R1 and IL-10R2 were also detectable in all the OSCC cell lines under serum-free conditions (data not shown).

### MISK81-5 squamous cell carcinoma cells are responsive to IL-22

**Figure 2.**
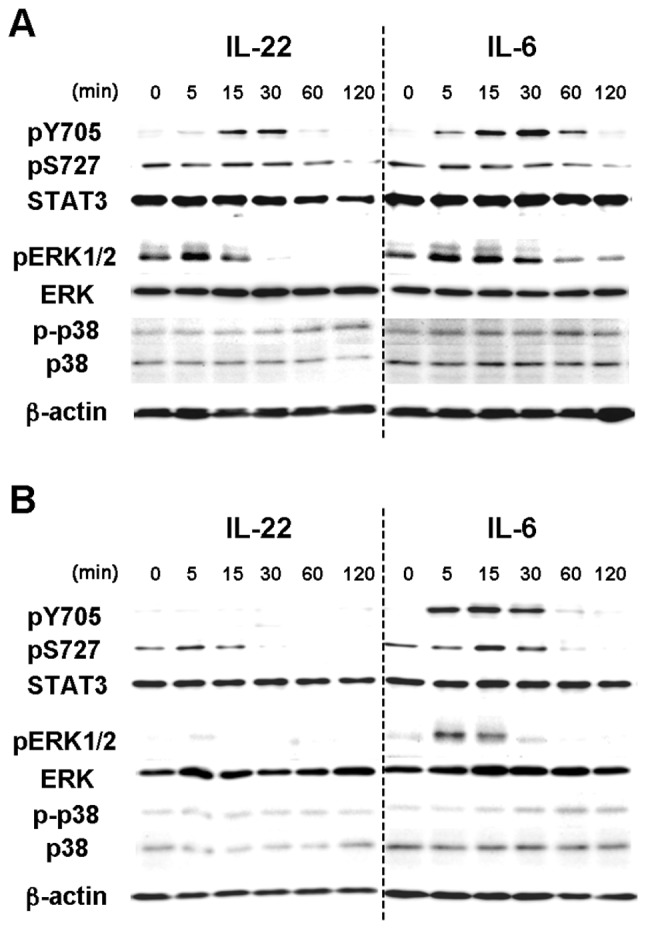
IL-22 induces transient STAT3 phosphorylation and the phosphorylation of several members of the MAPK pathways in MISK81-5, oral squamous cell carcinoma cells. (A) MISK81-5 and (B) HSC-3 cells were incubated with IL-22 (20 ng/ml) or IL-6 (20 ng/ml) for varying times up to 120 min. (A) IL-22 and IL-6 induced pY705-STAT3 in MISK81-5 cells within 15 and 5 min, respectively. IL-22 induced transient tyrosine phosphorylation of STAT3 in MISK81-5 cells with similar kinetics to IL-6. IL-22 and IL-6 induced subtle changes in pS727-STAT3 within the tested time periods. IL-22 induced a transient activation of ERK1/2 in MISK81-5 cells and also induced a delayed phosphorylation of p38 MAP kinase, similar to IL-6. (B) In HSC-3 cells, pY705-STAT3 was undetectable after IL-22 stimulation, although transient pY705-STAT3 expression was induced after IL-6 stimulation. IL-22 and IL-6 induced the transient serine phosphorylation of STAT3. HSC-3 cells stimulated with IL-6 showed activation of ERK1/2 and p38 MAP kinases similar to that in MISK81-5 cells. However, the activation in HSC-3 cells was undetectable after IL-22 treatment. All findings represent the results of three independent experiments.

IL-22 induced the tyrosine phosphorylation of STAT3 (pY705-STAT3) in MISK81-5 cells within 15 min, peaking at 30 min (Fig. 1B), as seen in other cell lines by IL-22 (13,17,24–26). This phosphorylation was transient, and decreased toward the baseline until reaching barely detectable levels after 120 min. The change in the serine phosphorylation of STAT3 (pS727-STAT3) in MISK81-5 cells treated with IL-22 was subtle within the tested periods. At the same time, pY705-STAT3 increased within 5 min and still remained detectable in MISK81-5 cells at least 1 h after IL-6 stimulation. IL-6 treatment led to a subtle change in pS727-STAT3 within the tested periods. Conversely, IL-6 had a similar effect on pY705-STAT3 in HSC-3 cells, but the activation of pY705-STAT3 by IL-22 was not detectable during the tested periods (Fig. 2A).

IL-22 induced the phosphorylation of ERK1/2 in MISK81-5 cells within 5 min, but the level slightly decreased at 15 min (Fig. 2B). This phosphorylation decreased to below control levels after 30 min. IL-22 also induced a delayed phosphorylation of p38 MAP kinase after 60 min. Although the peak of pERK1/2 was noted at 15 min, similar results were obtained in MISK81-5 cells treated with IL-6. The activation of ERK1/2 and p38 MAP kinases was undetectable in HSC-3 cells after IL-22 treatment (Fig. 2A). IL-6 showed similar activation of ERK1/2 and p38 MAP kinases to that in MISK81-5 cells treated with IL-22 or IL-6.

### IL-22 induces the translocation of pSTAT3 into the nucleus of MISK81-5 cells

**Figure 3.**
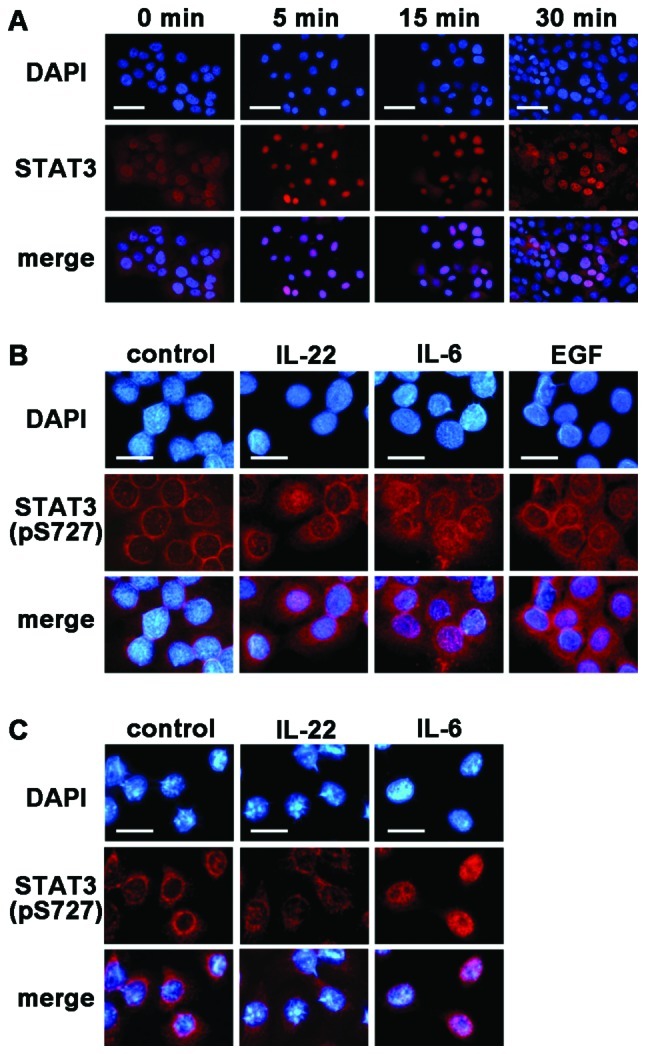
IL-22 translocates pSTAT3 into the nucleus in MISK81-5 cells. (A) MISK81-5 cells were either untreated as a control (left) or treated with IL-6. IL-6 led to the rapid translocation of most of the STAT3 into the nucleus of MISK81-5 cells within 5 min. After 30 min, STAT3 was noted in the cytoplasm again. (B) MISK81-5 or (C) HSC-3 cells were either untreated as a control (left) or treated with IL-22 or IL-6 for 30 min. (B) The translocation of pSTAT3 into the nucleus was noted in MISK81-5 cells treated with IL-22 as well as with IL-6 or EGF. (C) In HSC-3 cells, the translocation of pSTAT3 into the nucleus was not observed after IL-22 stimulation, but trans-location was induced by IL-6. Alexa Fluor^®^ IgG was used as the secondary antibody (red); nuclei were counter-stained with DAPI (blue); EGF was used as control for stimulating the activation of STAT3. (A) Scale bar, 30 *μ*m; (B and C) scale bar, 10 *μ*m.

STAT3 expression was noted in both the nucleus and cytoplasm of MISK81-5 cells before IL-6 stimulation, and was observed in the nucleus of many MISK81-5 cells within 5 min after IL-6 stimulation. STAT3 was again detectable in the cytoplasm after 30 min (Fig. 3A). The increased signal for pSTAT3 in the nucleus of MISK81-5 cells was observed at 30 min after IL-22 treatment (Fig. 3B), whereas no nuclear translocation of pSTAT3 was detected in HSC-3 cells treated with IL-22 (Fig. 3C).

### IL-22 promotes the expression of anti-apoptotic and mitogenic genes in MISK81-5 cells

Since cytokine stimulation can induce instability in the housekeeping gene expression (27–29), B2M was selected as the internal control among the various housekeeping genes tested using the geNorm system (http://medgen.ugent.be/~jvdesomp/genorm).

**Figure 4.**
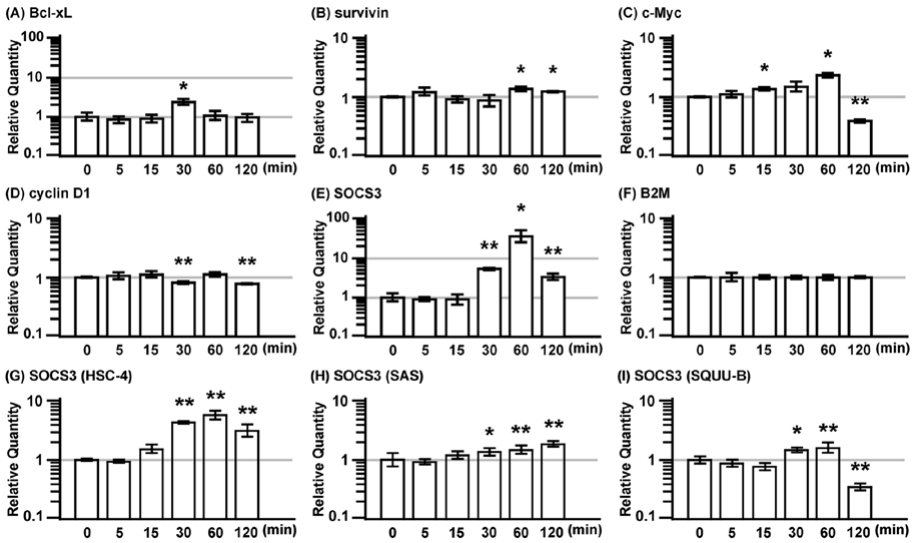
IL-22 transiently induces the expression of anti-apoptotic and mitogenic genes in MISK81-5 cells. MISK81-5 cells were treated with IL-22 (20 ng/ml) for various times as indicated. (A) Bcl-xL, (B) survivin, (C) c-Myc, (D) cyclin D1 and (E) SOCS3 were targeted as STAT3-downstream genes. Bcl-xL, c-Myc and SOCS3 exhibited a peak expression at 30, 60 and 60 min after IL-22 stimulation, respectively. However, c-Myc gene expression was significantly decreased at 120 min. IL-22 significantly increased the gene expression of survivin at 60 and 120 min and decreased cyclin D1 at 30 and 120 min. (F) B2M was used as a reference gene. The SOCS gene expression in (G) HSC-4, (H) SAS and (I) SQUU-B cells treated with IL-22 (20 ng/ml) for various times is shown as indicated. Significant differences between the stimulated and unstimulated samples are indicated with single or double asterisks (^*^p<0.05; ^**^p<0.01).

The expression of anti-apoptotic proteins, Bcl-xL and survivin, and the mitogenic proteins, c-Myc and cyclin D1, and the suppressor for STAT3, the SOCS3 gene, were examined in MISK81-5 cells treated with IL-22 (Fig. 4). The expression of Bcl-xL and c-Myc genes exhibited a 2.4-fold increase, and peaked at 30 and 60 min after IL-22 stimulation, respectively (Fig. 4A and C). However, the c-Myc gene expression dramatically decreased to 40% of the basal level at 120 min. SOCS3 expression was markedly induced at 30 min after IL-22 stimulation, it exhibited a 37-fold increase at 60 min and subsequently decreased at 120 min (Fig. 4E). IL-22 significantly increased the gene expression of survivin at 60 and 120 min. The expression of cyclin D1 significantly decreased at 30 and 120 min (Fig. 4B and D).

SOCS3 expression was markedly induced at 30 min in the HSC-4 cells after IL-22 stimulation, it peaked at 60 min, and subsequently decreased at 120 min (Fig. 4G). Similar results were observed for IL-22 stimulation in the SQUU-B cells. However, its expression dramatically decreased to less than the baseline level at 120 min (Fig. 4I). The SOCS3 expression in the SAS cells treated with IL-22 was gradually increased from 30 min to 120 min (Fig. 4H).

### IL-22 slightly induces tumor cell proliferation in vitro and the cellular NF-κB activation status

**Figure 5.**
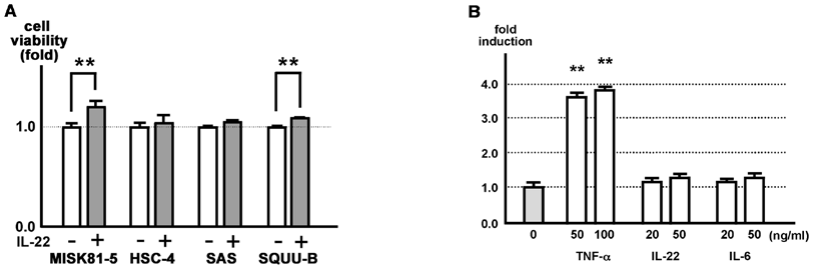
IL-22 affects MISK81-5 cell proliferation *in vitro*, but is negligibly associated with upregulation of the cellular NF-κB activation. (A) MISK81-5, HSC-4, SAS and SQUU-B cells were incubated with or without IL-22 (20 ng/ml) for 48 h. The viability of MISK81-5 and SQUU-B cells treated with IL-22 was increased by 1.3- and 1.1-fold in comparison to the control cells, respectively. A significant difference was noted between the stimulated and control samples (^**^p<0.01). (B) Stably transfected cells, MISK-pGL4-NF-κB cells were stimulated with TNF-α (50 or 100 ng/ml), IL-6 (20 or 50 ng/ml) or IL-22 (20 or 50 ng/ml) or with control medium. A luciferase assay was performed after 24 h. Each ratio is indicated as the relative expression compared to the unstimulated control samples, and indicates the mean ± SD (bars) of three values from one representative experiment. Significant differences in the transcriptional activity are indicated with double asterisks (^**^p<0.01).

The effect of IL-22 on the proliferation of MISK81-5, HSC-4, SAS and SQUU-B cells *in vitro* was examined. MISK81-5 and SQUU-B cells treated with IL-22 showed 1.3- and 1.1-fold increase in viability compared with control samples, respectively. A significant difference was demonstrated between the IL-22-treated cells and controls (p<0.01). Although HSC-4 and SAS cells were subtly increased by IL-22, there was no significant difference in the viability of these cells between the IL-22-treated cells and controls (Fig. 5A).

MISK-pGL4-NF-κB cells stimulated with 50 and 100 ng/ml TNF-α demonstrated significant 3.6-fold and 3.8-fold increases in luciferase activity, respectively, compared with the unstimulated cells (Fig. 5B). However, the effects of IL-22 and IL-6 were subtle or negligible. No significant difference was noted between the stimulated and control samples (Fig. 5B).

### IL-22 reduces the expression of keratinocyte differentiation-related genes

**Figure 6.**
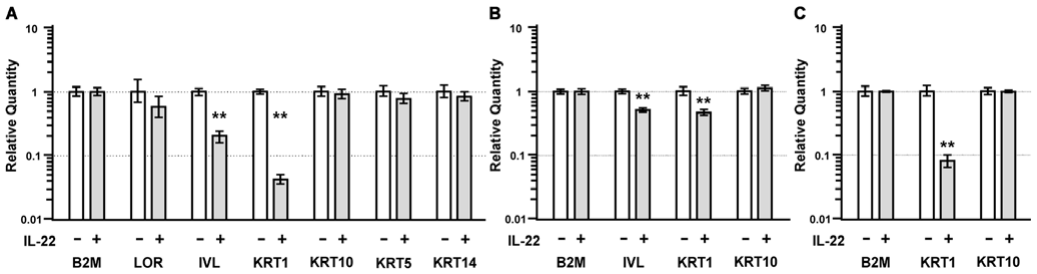
IL-22 treatment reduces the expression of keratinocyte differentiation-related genes. (A) The expression of loricrin (LOR), involucrin (IVL), keratin 1 (KRT1), keratin 10 (KRT10), keratin 5 (KRT5) and keratin 14 (KRT14) was compared between MISK81-5 cells stimulated with IL-22 for 24 h and unstimulated cells. (B) The expression of IVL, KRT1 and KRT10 was compared between HSC-4 cells stimulated with IL-22 for 24 h and unstimulated cells. (C) The expression of KRT1 was significantly decreased in the IL-22-treated SAS cells compared to that in unstimulated cells. Significant differences in the gene expression are indicated by double asterisks (^**^p<0.01).

The expression of the involucrin (IVL) and keratin 1 (KRT1) genes significantly decreased to ∼20% and ∼5% of control levels by IL-22 treatment, respectively (p<0.01; Fig. 6A). In addition, the expression of these genes in HSC-4 cells significantly decreased to ∼50% after IL-22 treatment (p<0.01) (Fig. 6B). The KRT1 expression in SAS cells significantly decreased to ∼10% after IL-22 treatment (p<0.01) (Fig. 6C). The expression of keratin 10 (KRT10) was unchanged in the MISK81-5 (Fig. 6A), HSC-4 (Fig. 6B), SAS (Fig. 6C) and SQUU-B cells treated with IL-22.

**Figure 7.**
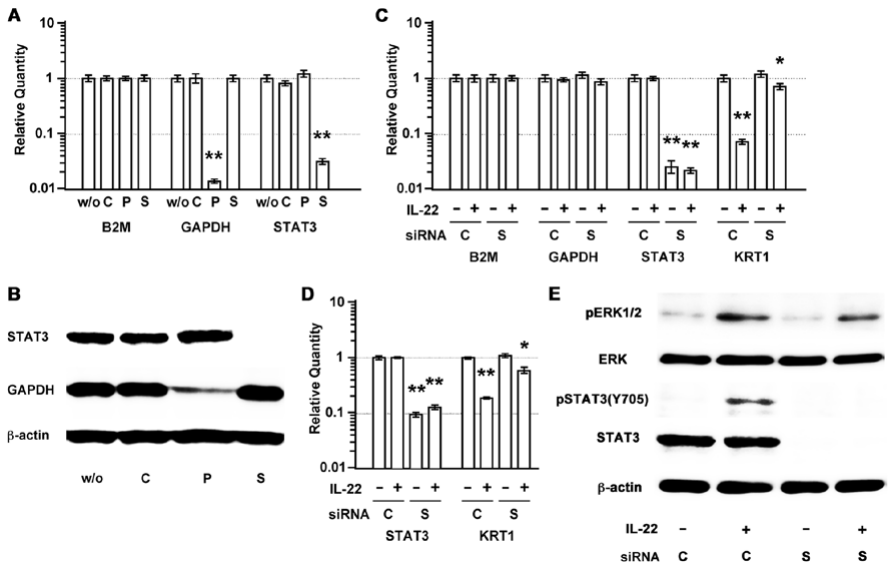
STAT3 siRNA inhibits IL-22-induced reduction of KRT1 expression, but it has little impact on pERK. (A) siRNA selectively reduced the gene expression in the MISK81-5 cells at 30 h after siRNA transfection. Significant differences in the gene expression are indicated by double asterisks (^**^p<0.01). B2M was used as a reference gene. (B) An immunoblot analysis also revealed that GAPDH and STAT3 siRNAs cause a depletion of the GAPDH and STAT3 protein levels in the MISK81-5 cells, respectively. (C) At 30 h before IL-22 stimulation, the MISK81-5 cells were transfected with siRNA. The expression of B2M, GAPDH, STAT3 and KRT1 was compared between MISK81-5 cells after IL-22 stimulation for 24 h and unstimulated cells (^*^p<0.05; ^**^p<0.01). (D) The downregulation of KRT1 expression by IL-22 was inhibited in the SAS cells transfected with a siRNA for STAT3 and in unstimulated cells. Significant differences in the gene expression are indicated by single or double asterisks (^*^p<0.05; ^**^p<0.01). (E) At 30 h after siRNA transfection, the MISK81-5 cells were treated with IL-22 for 10 min. Total cell lysates (10 *μ*g/sample) were analyzed by immunoblotting with an antibody against pY705-STAT3. The membrane was repeatedly reprobed and immunoblotted with an anti-pERK1/2, anti-total STAT3, or an anti-ERK antibody and then with an anti-β-actin antibody. w/o, sample without siRNA treatment; C, sample treated with negative control siRNA; P, sample treated with GAPDH siRNA as a positive control; S, sample treated with STAT3 siRNA.

To examine whether IL-22 induces a reduction of the KRT1 expression through STAT3, we used siRNA to selectively reduce the STAT3 expression. STAT3 siRNA induced a significant downregulation of the STAT3 mRNA and protein levels (Fig. 7A and B), and inhibited the downregulation of KRT1 expression by IL-22 (Fig. 7C). Similarly, the transfection of the SAS cells with a siRNA for STAT partially inhibited the downregulation of the KRT1 expression by IL-22 (Fig. 7D). However, neither ERK nor pERK1/2 was affected by the STAT3 siRNA treatment (Fig. 7E).

### IL-22 upregulates the expression of SERPINB3/4 (SCCA1/2) genes

**Figure 8.**
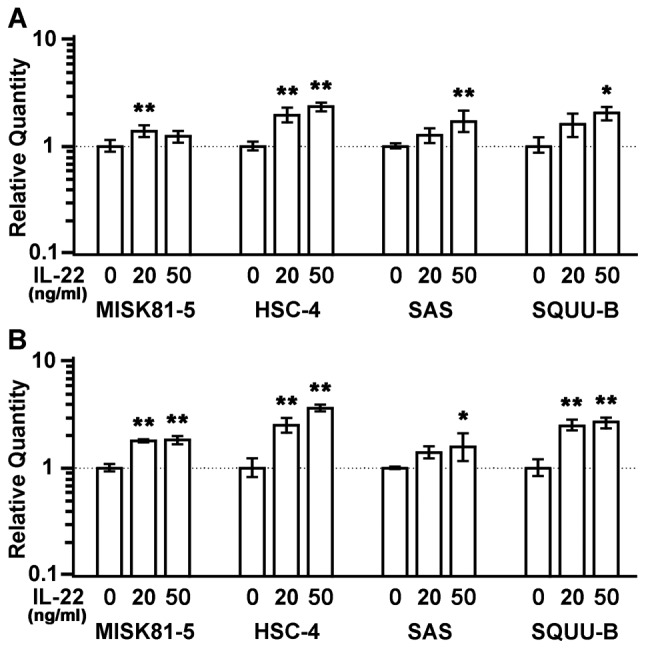
IL-22 treatment induces the upregulated expression of SERPINB3/4 genes in OSCC cells. The expression of the (A) SERPINB3 and (B) SERPINB4 genes was increased in the MISK81-5, HSC-4, SAS and SQUU-B cells stimulated with IL-22 (20 or 50 ng/ml) compared with those cultured in the control medium. Significant differences in the gene expression are indicated by single or double asterisks (^*^p<0.05; ^**^p<0.01).

Squamous cell carcinoma antigen (SCCA) 1 was originally identified in squamous cell carcinoma (SCC) of the uterine cervix (30). An elevated expression of SCCA1 and its isoform, SCCA2, has been used as a biomarker for aggressive SCC in the cervix, lung, head and neck (31–33). SCCA belongs to the serine protease inhibitor (Serpin) family of proteins, and SCCA1 and SCCA2 are called SERPNB3 and SERPINB4, respectively. The SERPINB3 expression showed respective 1.2-, 2.4-. 1.7- and 2.0-fold increases in the MISK81-5, HSC-4, SAS and SQUU-B cells treated with IL-22 (50 ng/ml) compared with control samples. The SERPINB4 expression also showed 1.8-, 3.6-. 1.6- and 3.0-fold increases, respectively, in the MISK81-5, HSC-4, SAS and SQUU-B cells treated with IL-22 (50 ng/ml) compared with control samples. A significant difference in the expression levels of these genes was noted between all of the IL-22-treated (50 ng/ml) cells and control cells, except for the SERPINB3 expression in MISK81-5 cells (p<0.01 or p<0.05) (Fig. 8).

## Discussion

Immunostaining for IL-22R revealed that the intensity was increased in the primary and metastatic OSCC cells. IL-22 induced the transient phosphorylation of STAT3 and led to its translocation into the nucleus. IL-22 activated the ERK and p38 MAPK pathways, but did not have a significant effect on NF-κB. IL-22 mildly affected the proliferation of OSCC cells and downregulated the expression of keratinocyte differentiation-related genes. STAT3 siRNA inhibited the IL-22-mediated downregulation of the keratinocyte differentiation-related genes, but did not affect the activation of the ERK pathway. The expression of the SERPINB3/4 genes in OSCC cells was upregulated by IL-22 stimulation, thus suggesting that IL-22 plays a key role in the biology of OSCC cells.

Immunohistochemical staining showed that IL-22R was expressed in OSCC. The expression of both IL-22 receptor chains was confirmed in MISK81-5, HSC-3, HSC-4, SAS and SQUU-B OSCC cell lines by RT-PCR. In the immunoblotting analysis, MISK81-5 cells showed the transient phosphorylation of STAT3 at Y705 by IL-22 stimulation. Similar results were reported for IL-22 stimulation in other types of cells (13,23). In the immunocytochemistry experiments, a transient translocation of STAT3 into the nucleus was observed in MISK81-5 cells. When pY705-STAT3 decreased, STAT3 was again detected in the cytoplasm, similar to unstimulated cells. These results suggest that pY705 mediated the translocation of pSTAT3. This finding was supported by the study of Zhong *et al*(34), in which the phosphorylation of STAT3 at Y705 was shown to lead to the translocation of STAT3 into the nucleus, thereby activating the transcription of multiple target genes. Conversely, the change in pS727-STAT3 was subtle in this study. The phosphorylation of STAT3 at S727 in OSCC cells was different from that in the study of Lejeune *et al*(13) who showed transient increases in pS727-STAT3 in hepatoma cells after treatment with IL-22. Although pS727 is thought to play a regulatory role in STAT3 activation, resulting in its maximal transcriptional activity (35), the function of pS727 remains unclear in this study. In addition, STAT3 phosphorylation was not observed in HSC-3 cells following IL-22 stimulation. This result indicates that the IL-22 receptors were functional in MISK81-5 cells, but that not all squamous cell carcinomas activate STAT3 signaling after exposure to IL-22.

The activity of MAP kinases such as ERK and p38 after IL-22 stimulation in this study (Fig. 2), is partly reminiscent of that in hepatoma cells observed in other studies (13,14). After IL-22 stimulation, ERK activation preceded that of STAT3. The phosphorylation of ERK1/2 induced by IL-22 stimulation was not affected by STAT3 siRNA (Fig. 7). These results suggest that other STAT3-independent mechanisms are acting on MISK81-5 cells under IL-22 stimulation. While IL-22 transiently activated ERK1/2 and induced a delayed phosphorylation of p38 MAP kinase, ERK1/2 phosphorylation decreased to less than the control level after 30 min. A similar result was seen in the IL-22 treatment of murine breast adenocarcinoma EMT6, in which ERK1/2 phosphorylation was inhibited by IL-22, thus leading to cell cycle arrest (17). Additionally, the transient activation of STAT3 also involved the transient upregulation of SOCS3 expression in OSCC cells. The transient upregulation of SOCS3 expression may affect the transient activation of STAT3 and STAT3-associated factors in OSCC cells, as SOCS3 acts as a suppressor of STAT signaling, while SOCS3 is one of the downstream genes of STAT3. IL-22 may constitutively contribute to the activation of STAT3 and the expression of anti-apoptotic and mitogenic genes in OSCC cells under the suppression of SOCS3, since SOCS3 causes growth inhibition in SCC cell lines (25,36). Indeed, IL-22 mildly stimulated the cell proliferation of MISK81-5 and SQUU-B cells in this study. The proliferation of HSC-4 and SAS cells was limited after IL-22 stimulation. This stimulation may be due to a complicated synergistic effect among the transiently increased activity of ERK1/2 and the expression of c-Myc and cyclin D1 genes, the inhibition of ERK1/2 phosphorylation, and/or SOCS3 expression.

Keratinocytes are thought to show changes in their expression and synthesis of cytoskeletal proteins after exposure to proliferative or inflammatory cytokines (37). IVL, LOR, KRT1 and KRT10 are the characteristic markers of normal suprabasal keratinocytes (38). IL-22 significantly reduced the expression of the IVL and/or KRT1 genes in MISK81-5, HSC-4 and SAS cells. Our results indicated that IL-22 could thus play a role in regulating the terminal differentiation of OSCC cells through STAT3 activation similar to the effects in keratinocytes. Since these factors play important roles during the terminal differentiation of keratinocytes and are associated with apoptotic processes (39–42), the control of the IL-22 function in OSCCs may therefore make it possible to induce apoptosis in OSCC cells via differentiation.

In this study, IL-22 induced the upregulation of SERPINB3 and SERPINB4 expression in OSCC cells. The downregulation of SERPINB3 by an antisense method significantly increased the cellular susceptibility to drug-induced apoptosis (43). Our previous study showed that SERPINB3/B4 contributed, at least in part, to preventing TNF-α induced cell death by impeding the cytochrome *c* release from the mitochondria (44). Ahmed *et al*(45) demonstrated that squamous carcinoma cells promote cell survival through activation of SERPINB3/B4 genes by activated STAT3. Thus, IL-22 may play a role in the attenuation of drug-induced apoptosis by the increasing the expression of SERPINB3/B4 in cancer cells.

Our present study shows that IL-22 affects several important functions of OSCC cells via the STAT3-dependent and/or -independent pathways, suggesting that IL-22 may play a role in carcinoma cell differentiation and the upregulation of SERPINB3/B4, well-known biomarkers for SCC. However, the response against IL-22 varies in OSCC cell lines. Further studies are required to elucidate the mechanisms by which IL-22 is involved in the biology of OSCC carcinogenesis. Elucidating the functions of IL-22 could lead to the development of new perspectives on this disease, and potentially new therapies with few side-effects, thereby improving the treatment of patients with OSCC.

## Figures and Tables

**Table I.** Primer sets used in the present study.

Target		Sequence
For semiquantitative RT-PCR		
IL-22R1	sense	5′-CTC CAC AGC GGC ATA GCC T-3′
	antisense	5′-ACA TGC AGC TTC CAG CTG G-3′
IL-10R2	sense	5′-GGC TGA ATT TGC AGA TGA GCA-3′
	antisense	5′-GAA GAC CGA GGC CAT GAG G-3′
β-actin	sense	5′-ATC TGG CAC CAC ACC TTC TAC AAT GAG CTG CG-3′
	antisense	5′-CGT CAT ACT CCT GCT TGC TGA TCC ACA TCT GC-3′
For quantitative RT-PCR		
Bcl-xL	sense	5′-TAG GGT GGC CCT TGC AGT TC-3′
	antisense	5′-GTG AGG CAG CTG AGG CCA TAA-3′
Survivin	sense	5′-TTC TCA AGG ACC ACC GCA TC-3′
	antisense	5′-GCC AAG TCT GGC TCG TTC TC-3′
c-Myc	sense	5′-CGG ATT CTC TGC TCT CCT CGA C-3′
	antisense	5′-CCT CCA GCA GAA GGT GAT CCA-3′
Cyclin D1	sense	5′-GTG CAT CTA CAC CGA CAA CTC CA-3′
	antisense	5′-TGA GCT TGT TCA CCA GGA GCA-3′
SOCS3	sense	5′-CCC AAG GAC GGA GAC TTC GAT-3′
	antisense	5′-GAA ACT TGC TGT GGG TGA CCA T-3′
TFRC	sense	5′-GCG AGC ACT GAC CAG ATA AGA ATG-3′
	antisense	5′-TCC CGA TAA TGT GTT AGG ATT GTG A-3′
β-actin	sense	5′-TGG CAC CCA GCA CAA TGA A-3′
	antisense	5′-CTA AGT CAT AGT CCG CCT AGA AGC A-3′
GAPDH	sense	5′-GCA CCG TCA AGG CTG AGA AC-3′
	antisense	5′-TGG TGA AGA CGC CAG TGG A-3′
B2M	sense	5′-CGG GCA TTC CTG AAG CTG A-3′
	antisense	5′-GGA TGG ATG AAA CCC AGA CAC ATA G-3′
Loricrin	sense	5′-TCA TGA TGC TAC CCG AGG TTT G-3′
	antisense	5′-TGC AAA TTT ATT GAC TGA GGC ACT G-3′
Involucrin	sense	5′-TAA CCA CCC GCA GTG TCC AG-3′
	antisense	5′-ACA GAT GAG ACG GGC CAC CTA-3′
Keratin 1	sense	5′-AGA TCA CTG CTG GCA GAC ATG G-3′
	antisense	5′-TGA TGG ACT GCT GCA AGT TGG-3′
Keratin 5	sense	5′-GAT AGC ATC ATC GCT GAG GTC AAG-3′
	antisense	5′-AGC CTC TGG ATC ATC CGG TTC-3′
Keratin 10	sense	5′-AGG CTG GCA GCT GAT GAC TTC-3′
	antisense	5′-CAG GGT CAG CTC ATC CAG CA-3′
Keratin 14	sense	5′-ACT TCA AGA CCA TTG AGG ACC TGA G-3′
	antisense	5′-CAG GGT CAG TTC GTC CAG CA-3′
SERPINB3	sense	5′-GGC AGC AAT ACC ACA TTG GTT C-3′
	antisense	5′-TGT ATT GCC TCA TCA TCT GTA TGG A-3′
SERPINB4	sense	5′-GGG ACT ATT GGC AAT GAT ACG ACA C-3′
	antisense	5′-AGG ACC TTG GCC TGT ACA TCC TC-3′
